# Do We Use Diamond Disks?

**Published:** 1878-05

**Authors:** 


### Editorial.

### "DO WE USE DIAMOND DISKS?"

Yes, in almost every case of proximate filling, and especially for dressing the edges and borders of almost all these cavities. Their greatest efficiency is not in separating teeth that are firmly in contact; such teeth should be slightly separated by wedging or by passing a thin separating saw between them; when this done it in a moment clears its way. It cuts truer, with greater rapidity than any other instrument with which we are familiar. Its cutting is so rapid that constant care must be exercised lest too much cutting is effected. It leaves a finished surface upon enamel or dentine.

As to durability we need only to refer to the fact that we have had one—made of nickel—in almost daily use during the last ten months, and it cuts now about as rapidly as at first.

It is an invaluable appliance in skillful hands, and a dangerous one in unskillful or careless hands. For properly using these or any other disks in the mouth, the Elliott engine, or one having its mode of motion, is almost a necessity while almost any of the other engines is as good and possibly better for using small burs, etc., the Elliott is infinitely better for disks.

